# Candida glabrata Fungemia Following Robotic Total Laparoscopic Hysterectomy and Bilateral Salpingo-oophorectomy in a Patient with Recurrent Vulvovaginitis: A Case Report

**DOI:** 10.7759/cureus.4349

**Published:** 2019-03-30

**Authors:** Hana F Mikdachi, Emily Spann

**Affiliations:** 1 Obstetrics and Gynecology, East Tennessee State University, Johnson City, USA

**Keywords:** fungemia, candida glabrata, hysterectomy, postoperative fever

## Abstract

Vulvovaginal candidiasis is a common gynecologic diagnosis that can be treated empirically with fluconazole. We present a patient that developed post-operative *Candida glabrata* (*C. glabrata*) fungemia after being empirically treated for vulvuovaginal candidiasis with fluconazole multiple times throughout the year prior to robotic total laparoscopic hysterectomy and bilateral salpingo-oophorectomy. *C. glabrata* is becoming increasingly resistant to azole antimycotic therapy. It is likely that this patient had undertreated fluconazole-resistant *C. glabrata* vulvovaginitis prior to surgery, and that the pelvic infection was the source of fungemia.

## Introduction

*Candida glabrata* (*C. glabrata*) is the second most common form of candida vulvovaginitis, after *Candida albicans *(*C. albicans*) [1,2]. Patients with candida vulvovaginitis are often empirically treated with fluconazole, the standard treatment for *C. albicans*. The majority of strains of *C. glabrata *are resistant to fluconazole, resulting in undertreatment of disease if treated empirically with fluconazole [3]. Preoperative pelvic infections such as bacterial vaginosis and trichomoniasis are known to increase risk of postoperative infections, but there is little documentation of postoperative infections resulting from vulvovaginal candidiasis [4]. We present a case of a patient who was empirically treated for candida vulvovaginitis with fluconazole three weeks prior to robotic total laparoscopic hysterectomy and bilateral salpingo-oophorectomy. She subsequently developed *C. glabrata* fungemia likely due to undertreated *C. glabrata *vulvovaginitis.

## Case presentation

We report a case of a 43-year-old nulliparous woman who was referred for surgical management of her abnormal uterine bleeding/heavy menstrual bleeding (AUB/HMB), which was treated with combined oral contraceptives until her diagnosis of estrogen receptor/progesterone receptor (ER/PR) positive ductal carcinoma in situ (DCIS), in her right breast at age 42. Combined oral contraceptive treatment was therefore discontinued which worsened her AUB/HMB. Her past medical history was also significant for autoimmune disorders including fibromyalgia, Sjogren syndrome and rheumatoid arthritis (RA) that presented following treatment of Hurthle cell thyroid cancer. Her RA was treated with sulfasalazine and hydroxychloroquine.

When she presented to our clinic for management of her AUB/HMB, her DCIS of the breast was treated with a lumpectomy and radiotherapy and she was about to begin tamoxifen therapy. A hysterectomy was determined to be the best management option for her AUB/HMB as she was unable to use hormonal management, had multiple risk factors for endometrial cancer including diabetes mellitus and class III obesity, and was about to begin tamoxifen therapy which would also increase her risk for endometrial cancer. A bilateral salpingo-oophorectomy was recommended by her oncologist to decrease her risk of recurrence of breast cancer. She was scheduled for a robotic total laparoscopic hysterectomy and bilateral salpingo-oophorectomy (RTLH/BSO). Throughout the year prior to surgery she was empirically treated with fluconazole for vulvovaginal candidiasis on several occasions. Her last treatment was three weeks prior to surgery, which resolved her symptoms.

Her RTLH/BSO was uncomplicated and she tolerated the procedure well. She was discharged home on the same day in a stable condition. She presented to the emergency department on post-operative day three with fevers, tachycardia, headaches and nausea. On physical exam, erythema and edema were noted around the supraumbilical incision site with no drainage or separation of the skin. There was no evidence of a deep seroma, hematoma, crepitus, or peritonitis. Computed tomography (CT) imaging of the abdomen and pelvis showed no abnormal findings. Chest X-ray showed mild atelectasis. The infectious diseases team was consulted, and treatment for suspected cellulitis was initiated with vancomycin and piperacillin/tazobactam. Blood culture and antistreptolysin O titer were collected. Overnight, her temperature and white blood cell count (WBC) trended down but remained elevated. The general surgery team was consulted, and their assessment did not indicate an intrabdominal process. They believed the fevers were driven by the surgical site infection and recommended that her erythematous incision be opened to assess for possible abscess or hematoma and to assess the integrity of the fascia. This was performed, and her incision drained mostly serosanguinous fluid with no purulent matter. The fascia was intact.

Given her continued febrile episodes, the infectious diseases team exchanged piperacillin/tazobactam for meropenem. Her RA home medications were restarted to assure that a RA flare up was not a source of her fevers.

Her severe headaches did not respond to her home triptan medication or ketorolac. The neurology team was consulted, and a magnetic resonance imaging (MRI) of her brain was ordered, which did not reveal any acute process.

At the end of the third day of hospitalization, the blood cultures grew *C. glabrata*. Intravenous (IV) anidulafungin was initiated. After the addition of antifungals, her vital signs improved, and WBC dropped to normal range. Her headaches resolved. Vancomycin and meropenem were continued as treatment for her surgical site cellulitis. She eventually defervesced on post-operative day 10. She was continued on antifungals and antibiotics via peripherally inserted central catheter (PICC) for four weeks.

Consent was obtained by the patient to use this case for publication.

## Discussion

We report a case of *C. glabrata* fungemia occurring following RLTH/BSO; *C. glabrata *was likely introduced from her vagina at the time of her hysterectomy. *C. glabrata* is the second most common form of candida vulvovaginitis, after *C. albicans *[2,5]. *C. glabrata* is increasing in incidence as a cause of fungemia in the United States, and is second only to *C. albicans* as source of fungemia [6]. Most cases of *C. glabrata* fungemia are seen in immunocompromised and hospitalized patients, such as human immunodeficiency virus (HIV) infected, neutropenic, and critical care patients [1,5,7].

Pre-operative pelvic infections can lead to post-operative complications. Bacterial vaginosis or trichomoniasis vaginitis increases a patient’s risk for post-hysterectomy vaginal cuff cellulitis or abscess [4]. There is very little documentation of pre-operative infections with vulvovaginal candidiasis leading to fungemia, as in our patient.

The recommended treatment of uncomplicated vulvovaginal candidiasis is a single 150 mg dose of fluconazole or topical antifungals [3]. Patients, including the one in this case, are often empirically treated through a clinical diagnosis. Our patient presented with these symptoms on multiple occasions and was repeatedly treated empirically with fluconazole with improvement of her symptoms. Studies have shown that women with recurrent vulvovaginal candidiasis can find relief with fluconazole but then experience recurrences. These patients may find relief with maintenance fluconazole for suppression [8].

In cases of recurrent vulvavaginal candidiasis, it is appropriate to determine the strain of candidiasis and to do susceptibility studies to determine the optimal treatment. This is especially important in the setting of an immunocompromised patients. One prospective observational study highlighted the importance of fungal cultures to diagnose and to select the appropriate treatment in patients with vulvovaginal candidiasis, given the increasing prevalence of non-albicans species [9]. An analysis of vaginal flora in women with recurrent vulvovaginal candidiasis found that these women had a higher percentage of non-albicans species, including *C. glabrata* [10]. This information should increase the clinician’s suspicion for glabrata or another non-albicans species as the source of the recurrent infection.

The CDC has three recommendations for* C. glabrata *vulvovaginitis that is unresponsive to oral azoles. These are: topical intravaginal boric acid in gelatin capsule, 600 mg daily for 14 days; nystatin intravaginal suppositories, 100,000 units daily for 14 days; topical 17% flucytosine cream alone or in combination with 3% Amphotericin B cream daily for 14 days [3].

In our case, the empiric treatment with fluconazole did not effectively treat the vuluvovaginal candidiasis. IV anidulafungin, an echinocandin, was necessary to treat the resulting fungemia. A review of susceptibility of blood stream infections of Candida species in the United States from 1997 to 1999 showed a decreasing susceptibility of *C. glabrata* to fluconazole [11]. A retrospective cohort study of *C. glabrata* fungemia treatment with fluconazole versus echinocandin found that both treatments were effective, but echinocandins were independently associated with complete response. Trends also indicated that echinocandins were more appropriate treatment for more ill patients, although this finding was not statistically significant. Antifungal choice did not have an effect on mortality [12]. Another review of randomized controlled trials of the treatment of invasive candidiasis found that there was decreased mortality with echinocandin use over fluconazole therapy [13].

Many recent studies have highlighted the importance of susceptibility testing in cases of fungemia. Increasing resistance to echinocandins and azoles has been identified in cases of fungemia, especially with *C. glabrata*. Some have advised that prescribing these medications should be done more judiciously to prevent increasing resistance [14]. Others have suggested that the use of azoles in cases of *C. glabrata* fungemia should not be done in the absence of susceptibility testing [11].

## Conclusions

Following the review of this case and the relevant literature, we wish to emphasize the importance of determining the strain of candidiasis and doing susceptibility studies in patients with recurrent vulvovaginal candidiasis that does not respond to empiric therapy prior to any surgical interventions. This is especially important in the preoperative setting to prevent fungemia, as in our case.

## References

[1] Bross J, Talbot GH, Maislin G, Hurwitz S, Strom BL (1989). Risk factors for nosocomial candidemia: a case-control study in adults without leukemia. Am J Med.

[2] Makanjuola O, Bongomin F, Fayemiwo SA (2018). An update on the roles of non-albicans Candida species in vulvovaginitis. J Fungi.

[3] Pappas PG, Kauffman CA, Anders DR (2016). Clinical practice guideline for the management of candidiasis: 2016 update by the Infectious Diseases Society of America. Clin Infect Dis.

[4] Soper DE, Bump RC, Hurt WG (1990). Bacterial vaginosis and trichomoniasis vaginitis are risk factors for cuff cellulitis after abdominal hysterectomy. Am J Obstet Gynecol.

[5] Fidel PL, Vazquez JA, Sobel JD (1999). Candida glabrata: review of epidemiology, pathogenesis, and clinical disease with comparison to C. albicans. Clin Microbiol Rev.

[6] Pfaller MA, Diekema DJ (2007). Epidemiology of invasive candidiasis: a persistent public health problem. Clin Microbiol Rev.

[7] Beck-Sague C, Jarvis WR (1993). Secular trends in the epidemiology of nosocomial fungal infections in the United States, 1980-1990. National Nosocomial Infections Surveillance System. J Infect Dis.

[8] Crouss T, Sobel JD, Smith K, Nyirjesy P (2018). Long term outcomes of women with recurrent vulvovaginal candiasis after a course of maintenance antifungal therapy. J Low Genit Tract Dis.

[9] Nyirjesy P, Seeney SM, Terry MH, Jordan CA, Buckley HR (1995). Chronic fungal vaginitis: the value of cultures. Am J Obstet Gyencol.

[10] Sobel JD (1992). Pathogenesis and treatment of recurrent vulvovaginal candidiasis. Clin Infect Dis.

[11] Pfaller Pfaller, Diekema DJ, Jones RN (2001). International surveillance of bloodstream infections due to candida species: frequency of occurrence and in vitro susceptibilities to fluconazole, ravuconazole, and voriconazole of isolates collected from 1997 through 1999 in the SENTRY antimicrobial surveillance program. J Clin Microbiol.

[12] Eschenauer GE, Carver PL, Lin SW (2013). Fluconazole versus echinocandin for Candida glabrata fungemia: a retrospective cohort study. J Antimicrob Chemother.

[13] Andes DR, Safdar N, Baddley JW (2012). Impact of treatment strategy on outcomes in patients with candidemia and other forms of invasive candidiasis: a patient-level quantitative review of randomized trials. Clin Infect Dis.

[14] Pappas PG, Lionakis MS, Arendrup MC, Ostrosky-Zeichner L, Kullberg BJ (2018). Invasive candidiasis. Nat Rev Dis Primers.

